# Identification of the MADS-Box Gene Family and the Key Role of *BrAGL27* in the Regulation of Flowering in Chinese Cabbage (*Brassica rapa* L. ssp. *pekinensis*)

**DOI:** 10.3390/ijms26062635

**Published:** 2025-03-14

**Authors:** Xinyu Gao, Yang Li, Yun Dai, Xiangqianchen Li, Can Huang, Shifan Zhang, Fei Li, Hui Zhang, Guoliang Li, Rifei Sun, Huanzhong Song, Li Zhang, Zhendong Chen, Shujiang Zhang

**Affiliations:** 1 State Key Laboratory of Vegetable Biobreeding, Institute of Vegetables and Flowers, Chinese Academy of Agricultural Sciences, Beijing 100081, China; gaoxy7078@163.com (X.G.); daiyun1210@126.com (Y.D.); lixiangqianchen@163.com (X.L.); 15511256019@163.com (C.H.); zhangshifan@caas.cn (S.Z.); lifei@caas.cn (F.L.); zhanghui05@caas.cn (H.Z.); liguoliang@caas.cn (G.L.); sunrifei@caas.cn (R.S.); 2 Guangxi Academy of Agricultural Sciences, 174 East University Road, Nanning 530007, China; liyang@gxaas.net (Y.L.); songhuanzhong@gxaas.net (H.S.); nkyzl@gxaas.net (L.Z.)

**Keywords:** *BrAGL27*, Chinese cabbage, MADS-box gene family, vernalization

## Abstract

Chinese cabbage (*Brassica rapa* L. ssp. *pekinensis*) is a key vegetable crop in Asia, but its commercial value is often reduced by premature flowering triggered by vernalization. The molecular mechanisms behind this process are not fully understood. MADS-box genes, as crucial transcriptional regulators, play vital roles in plant development, including flowering. In this study, 102 MADS-box genes were identified in Chinese cabbage through bioinformatics analyses, covering phylogeny, chromosomal localization, and gene structure. Real-time quantitative PCR and RNA-seq data analysis revealed that the expression level of AGL27 declined as vernalization time increased. To determine *BrAGL27*′s functions, we obtained BrAGL27-overexpressed (OE) *Arabidopsis thaliana* lines that showed significantly later flowering compared with the wild type (WT). The expression levels of flowering suppressor genes *AtFLC* and *AtTEM1* were significantly high-regulated in the BrAGL27-OE lines compared to WT plants, while the expression levels of the floral genes *AtSPL15*, *AtSOC1*, *AtFT*, and *AtAP3* were significantly lower in the BrAGL27-overexpressed lines than in the wild type. These findings enhance understanding of MADS-box genes in vernalization and flowering regulation, offering a basis for further research on bolting resistance and flowering control in Chinese cabbage.

## 1. Introduction

Chinese cabbage (*Brassica rapa* L. ssp. *pekinensis*), also known as heading or wrapping cabbage, is a leafy vegetable from the cruciferous family. This versatile crop has been a staple in China’s agricultural heritage for centuries [1]. Among *Brassica* vegetables, it is one of the most economically significant, particularly across Asia [2]. Known for its rapid growth and development, Chinese cabbage is vital to agricultural production. However, during spring cultivation, it is highly susceptible to vernalization conditions that can trigger premature bolting and flowering, reducing its commercial value. In contrast, vernalization is beneficial in breeding programs, where it accelerates the development of superior varieties [1].

Research has shown that flowering time in Chinese cabbage is strongly influenced by environmental factors, especially the interaction between shoot apex activity and vernalization [3]. Prolonged cold exposure, or vernalization, is essential for flowering in many temperate plants [2]. This cold-induced transition from vegetative to reproductive growth is particularly critical for biennial and winter annual crops [4].

MADS-box genes play a central role in plant development, particularly in the complex regulation of flower formation. The term “MADS” is derived from four key members of this gene family identified in fungi, plants, and animals: MCM1 in yeast, AGAMOUS in *Arabidopsis thaliana*, DEFICIENS in snapdragon, and SERUM RESPONSE FACTOR in humans [5,6,7,8,9]. These MADS-box proteins are pivotal regulators of flowering time, controlling the reprogramming of meristem cell fate in shoot apical meristems [10]. As transcription factors, MADS-box genes regulate a wide array of developmental processes in plants [11]. While their principal activities are in flower and fruit development [12,13], several MADS-box genes are expressed in vegetative tissues, ovules, and embryos, demonstrating their multifaceted significance in plant development [14,15,16,17]. To date, MADS-box genes have been identified and classified in several dicot and monocot plants, including *Arabidopsis thaliana* [18], *Vitis vinifera* [19], cucumber [20], banana [21], wheat [22], soybean [23], and Chinese jujube [24]. In *Arabidopsis thaliana*, 107 MADS-box genes have been identified, with their functions extensively studied [18,25]. Now, the pan-genome of Chinese cabbage has been established [26]. Despite the fact that Chinese cabbage and *Arabidopsis thaliana* are both part of the Brassicaceae family and share a relatively close genetic relationship, with many gene functions potentially being similar, there still exist notable differences in the regulatory networks and mechanisms governing the flowering genes between the two species [27]. For example, the expression of *AtFLC* is specific in different tissues and developmental stages of *Arabidopsis thaliana.* During the vegetative growth stage, *AtFLC* is expressed in tissues such as the shoot apex and leaves, inhibiting flowering. As the vernalization process proceeds, the expression of *AtFLC* in the shoot apical meristem gradually decreases, preparing for flowering. The expression pattern of *FLC* in Chinese cabbage has some similarities with that in *Arabidopsis thaliana*, but there are also differences. During the vegetative growth stage of Chinese cabbage, *BrFLC* is highly expressed in parts such as the leaves and growing points, inhibiting premature flowering. However, due to the special developmental process of head formation in Chinese cabbage, the expression of *BrFLC* during the head formation period may be specially regulated to coordinate the relationship between head growth and flowering [28]. Therefore, some MADS-box genes’ functions in Chinese cabbage remain insufficiently understood.

To address this gap, a systematic analysis of the MADS-box genes in Chinese cabbage was conducted, encompassing their gene structure, conserved motifs, phylogenetic relationships, chromosomal localization, and expression patterns across various tissues under different durations of vernalization treatment. These results, coupled with RNA-seq data, provide a foundation for selecting specific MADS-box genes for further investigation. For instance, subcellular localization and overexpression analysis suggest that *BrAGL27*, a member of the MADS-box gene family, functions as a flowering suppressor. These findings offer valuable insights for future research on the molecular mechanisms underlying flowering regulation in Chinese cabbage. Therefore, we want to further explore the role that *BrAGL27* plays in the flowering of Chinese cabbage and how it affects the flowering process of Chinese cabbage.

## 2. Results

### 2.1. Chromosomal Location Analysis of MADS-Box Genes

**Figure 1:**
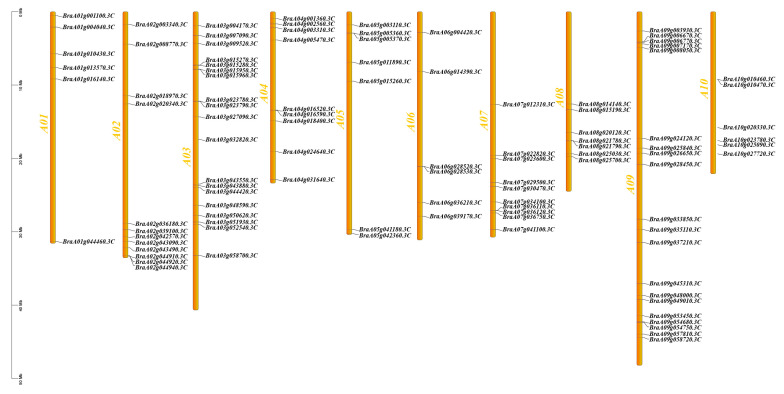
Schematic representation of the chromosomal distribution of the MADS-box genes in Chinese cabbage: a total of 102 MADS-box genes were mapped across 10 chromosomes (chr1–chr10).

The chromosomal distribution of MADS-box genes in Chinese cabbage was analyzed using genomic annotation data. A total of 102 MADS-box genes were identified, distributed across all ten chromosomes (Figure 1). These genes were predominantly clustered near the distal ends of the chromosomes, with significantly fewer genes located in the central regions. This uneven distribution may reflect functional or regulatory distinctions associated with their genomic positioning.

### 2.2. Gene Duplication and Synteny Analysis of the MADS-Box Family in Chinese Cabbage

**Figure 2:**
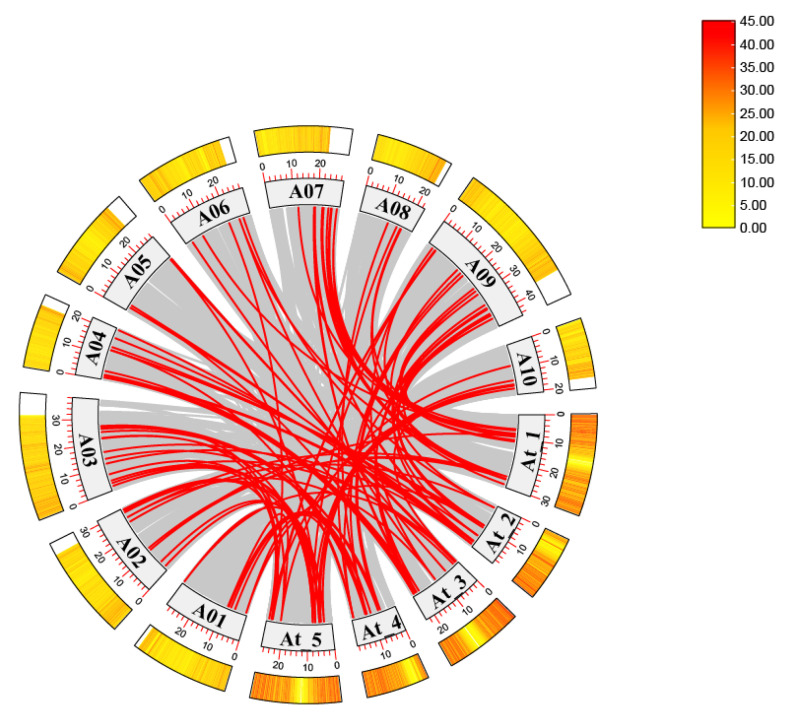
Schematic representation of the chromosomal distribution and two interchromosomal relationships of the MADS-box gene family between Chinese cabbage and *Arabidopsis thaliana*. Red lines highlight duplicated MADS-box gene pairs, with chromosome numbers labeled at the base of each chromosome. The numerical values on the right represent the FPKM (fragments per kilobase of transcript per million mapped reads) values.

The chromosomal distribution of MADS-box genes in Chinese cabbage was analyzed, revealing that the 102 genes were randomly distributed across its 10 chromosomes (Figure 2). To further investigate evolutionary relationships, comparative synteny maps were constructed between Chinese cabbage and *Arabidopsis thaliana*. The outer circle of the graph is composed of multiple arc-shaped strips labeled with numbers (such as A01–A10, At 1–At 5). These strips represent the chromosomes of Chinese cabbage and *Arabidopsis thaliana*. The scale and color gradient on each strip indicate the physical position of the chromosome and the gene density. The redder the color, the higher the gene density at this position. A large number of red and gray lines in the graph connect different chromosome strips, and these lines represent collinearity relationships. Red lines represent strong collinearity relationships, indicating that there are similar gene arrangements or homologous sequences in the connected chromosome regions of different species. Gray lines represent relatively weak collinearity relationships. There are a total of 56 red lines in the graph, indicating that there are 56 strong collinearity relationships between Chinese cabbage and *Arabidopsis thaliana*, and there are similar gene arrangements or homologous sequences in the chromosomal regions of these two species (Figure 2).

### 2.3. Phylogenetic Analysis of Chinese Cabbage MADS-Box Proteins

**Figure 3:**
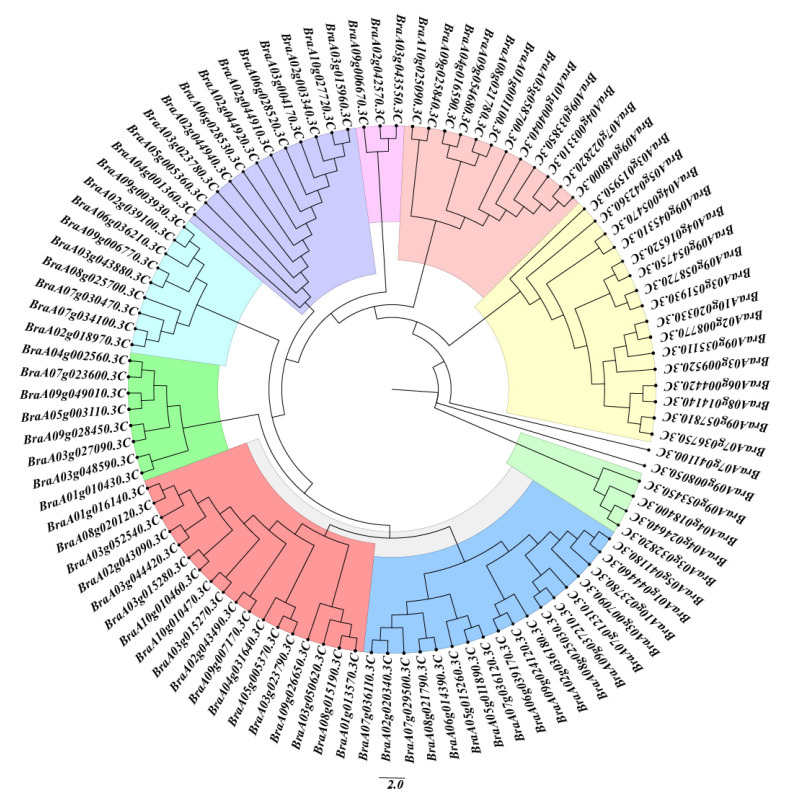
Phylogenetic analysis of Chinese cabbage MADS-BOX genes. A total of 102 MADS-box proteins were grouped into nine distinct phylogenetic subgroups.

A total of 102 MADS-box gene sequences were identified for the first time in the *Brassica rapa* genome. The full-length coding sequences of the MADS-box genes ranged from bp 189 (*BraA07g036120.3C*) to bp *2259* (*BraA01g015320.3C*) (Supplementary Table S1). The theoretical isoelectric point (pI) of the MADS-box proteins ranged from 4.86 (*BraA03g032820.3C*) to 10.11 (*BraA07g036120.3C*) (Supplementary Table S2). A phylogenetic tree was constructed using the neighbor-joining method to explore the evolutionary relationships among MADS-box proteins in Chinese cabbage. The analysis grouped the 102 MADS-box proteins into nine distinct phylogenetic subgroups (Figure 3).

### 2.4. Analysis of Conserved Motifs, Structural Domains, and Gene Structure in Chinese Cabbage MADS-Box Genes

**Figure 4:**
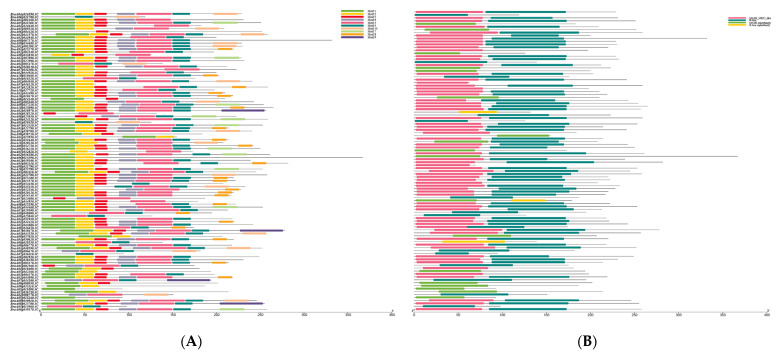
Characterization of the conserved motifs and conserved structural domains of the MADS-box gene family. (**A**) MeMe motifs. (**B**) Conserved domains.

The conserved motifs and structural domains of the Chinese cabbage MADS-box gene family were analyzed using TBtools (v 2.154) [29]. MEME [30] was employed to identify conserved motifs in the MADS-box protein sequences, revealing a total of 10 motifs. Additionally, a detailed analysis of 102 MADS-box protein sequences was performed, identifying four conserved domains encoded by the MADS-box gene family (Figure 4). These findings highlight the structural complexity and functional diversity of MADS-box genes in Chinese cabbage.

### 2.5. Heat Map of MADS-Box Gene Expression Under Different Vernalization Treatments

**Figure 5:**
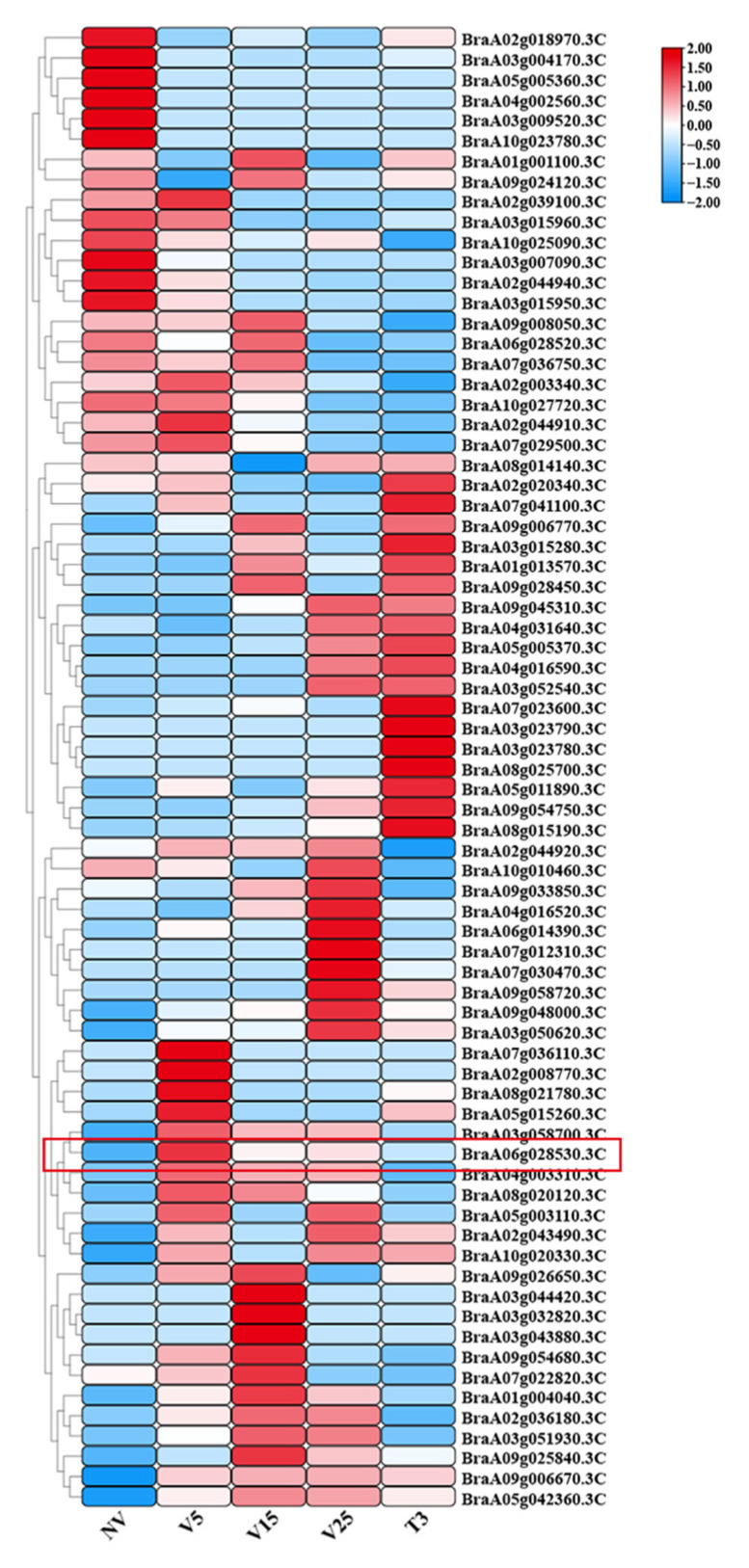
Expression of MADS-box genes under different vernalization time treatment. The numerical values on the right represent the FPKM (fragments per kilobase of transcript per million mapped reads) values.

Significant molecular-level shifts were observed across various samples and genes under different vernalization conditions. To investigate the expression patterns of genes regulating bolting and flowering, a heat map was constructed to visualize their expression levels. NV represents ‘non-vernalized,’ while V5, V15, and V25 correspond to 5, 15, and 25 days of vernalization, respectively. T3d indicates plants that underwent a 25-day vernalization treatment followed by a 3-day recovery period. Notably, the expression of *BrAGL27* decreased with increasing vernalization time, suggesting its potential role as a flowering inhibitor (Figure 5).

### 2.6. Expression Patterns of the BrAGL27 Gene in Response to Vernalization

**Figure 6:**
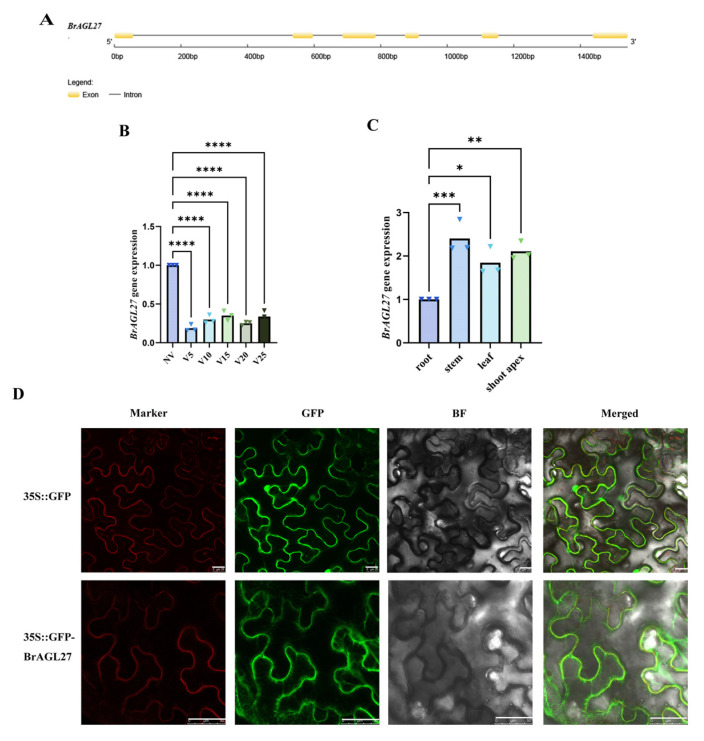
Preliminary analysis of *BrAGL27* expression patterns in Chinese cabbage. (**A**) Genomic structure of *BrAGL27*. (**B**) qRT-PCR analysis of *BrAGL27* expression across different vernalization stages. Asterisks indicate significant differences between vernalization treatments (**** *p* < 0.0001). (**C**) qRT-PCR analysis of *BrAGL27* expression in various plant tissues in response to vernalization. Asterisks indicate significant differences (* *p* < 0.05, ** *p* < 0.01, *** *p* < 0.001). (**D**) Subcellular localization of BrAGL27-GFP: *Agrobacterium tumefaciens* was used to transiently express the BrAGL27-GFP protein (or GFP) and a cell membrane marker protein (mCherry) in tobacco epidermal cells. The merged image combines GFP fluorescence, marker signals, and bright-field images. Scale bars = 25 μm/50 μm.

The *BrAGL27* gene encodes a MADS-box protein that plays a key role in the flowering process of plants. In Chinese cabbage, its coding sequence is 471 bp in length and consists of six exons and five introns (Figure 6A).

To further refine the analysis of *BrAGL27* expression at different vernalization time points, we conducted expression level measurements of *BrAGL27* across various stages of vernalization. Notably, the expression of *BrAGL27* showed a trend of initial decrease followed by an increase, starting from the non-vernalized (NV) stage and extending to 25 days of vernalization (V25) (Figure 6B). Further analysis of its relative expression in different tissues—roots, stems, leaves, and the shoot apex—revealed higher expression levels in stems, leaves, and the shoot apex compared to roots under vernalization treatment (Figure 6C).

Subcellular localization studies were conducted to determine the cellular location of the BrAGL27 protein. Cells expressing the BrAGL27-GFP fusion protein exhibited distinct luminescence primarily at the cell membrane, whereas GFP fluorescence was uniformly distributed in all regions of the control tobacco cells transformed with an empty vector (Figure 6D). These findings indicate that BrAGL27 is predominantly localized to the cell membrane.

### 2.7. Overexpression of BrAGL27 Results in a Late-Flowering Phenotype in Plants

**Figure 7:**
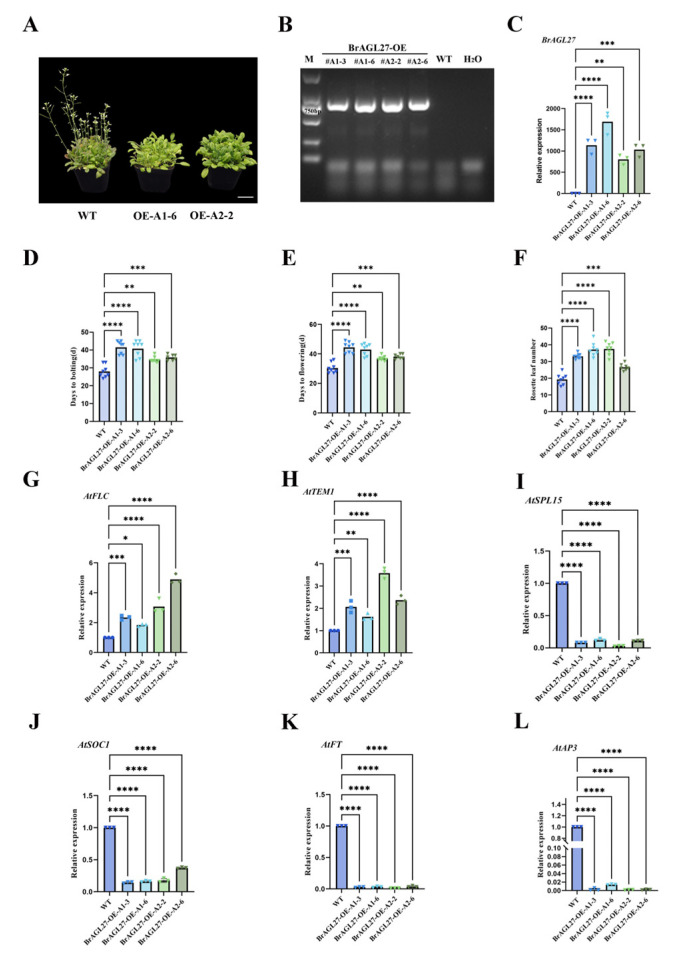
Analysis of the molecular function of *BrAGL27* in Chinese cabbage. (**A**) Phenotypes of *BrAGL27*-OE-A1-6, *BrAGL27*-OE-A2-2, and WT lines after 35 d of growth on medium. White bar at bottom right corner = 3 cm. (**B**) PCR amplification of *BrAGL27* coding sequences from *BrAGL27*-OE lines. (**C**) Relative expression levels of *BrAGL27* in WT and *BrAGL27*-OE lines. Error bars represent standard deviation (*n* = 3). (**D**–**F**) Comparison of bolting time, flowering time, and rosette leaf number between WT and *BrAGL27*-OE lines. Error bars represent standard deviation (*n* = 8). (**G**–**L**) Relative expression levels of *AtFLC*, *AtTEM1, AtSPL15*, *AtSOC1, AtFT*, and *AtAP3* in WT and *BrAGL27*-OE lines. Error bars represent standard deviation (*n* = 3). The findings are shown as mean ± standard deviation, with the following significance values: * *p* ≤ 0.05; ** *p* ≤ 0.01; *** *p* ≤ 0.001; **** *p* ≤ 0.0001; and non-significant (ns) (*p* > 0.05).

To investigate the function of *BrAGL27*, we overexpressed it in wild-type (WT) *Arabidopsis thaliana* (Figure 7B). Transgenic *Arabidopsis thaliana* plants constitutively expressing *BrAGL27* were generated using the pCAMBIA1305-*BrAGL27* vector and confirmed through molecular identification. The growth and developmental characteristics of the transgenic lines and WT plants were monitored throughout their life cycle. The overexpression of *BrAGL27* resulted in slower growth and a significant delay in flowering compared to WT plants. Specifically, the transgenic lines bolted approximately seven days later than the WT plants (Figure 7A).

Additionally, key growth metrics, including the number of rosette leaves at bolting, days to bolting, and days to flowering, were recorded for the pCAMBIA1305-*BrAGL27* lines and WT plants (Figure 7G–L). Transgenic plants exhibited around 10 additional rosette leaves compared to WT plants. Expression analysis confirmed that *BrAGL27* levels in the overexpression (*BrAGL27*-OE) lines were significantly higher than in WT plants (Figure 7F).

Importantly, the expression of flowering suppressor genes *AtFLC* and *AtTEM1* were significantly high-regulated in the BrAGL27-OE lines compared to WT plants (Figure 7G–H), while *AtSPL15*, *AtSOC1*, *AtFT,* and *AtAP3* were significantly downregulated in the BrAGL27-OE lines compared to WT plants (Figure 7I–L). Since *AtFLC* and *AtTEM1* are known inhibitors of flowering, *AtSPL15*, *AtSOC1*, *AtFT*, and *AtAP3* are floral genes; their reduced and increased expression in *BrAGL27*-OE lines suggests that *BrAGL27* interacts with these pathways to regulate flowering time. These results indicate that the heterologous expression of *BrAGL27* delays flowering by modulating key flowering suppressor and promoter genes.

## 3. Discussion

MADS-box genes regulate a wide range of biological processes in plants, including vegetative and reproductive growth. They play critical roles in inflorescence, flower, and fruit development [9,31,32,33]. Comparative genomic analyses have revealed that Chinese cabbage underwent genome triplication following its divergence from *Arabidopsis thaliana* [27]. The genome of Chinese cabbage is significantly more complex than that of *Arabidopsis thaliana*. However, no comprehensive or systematic studies on the MADS-box gene family in Chinese cabbage have been conducted. This study aimed to identify and analyze the MADS-box gene family in Chinese cabbage, advancing our understanding of their functions. *Arabidopsis thaliana* was the first dicot to have its entire genome sequenced and made publicly available, marking a milestone in plant genetics [34]. Subsequently, the genome of Chinese cabbage was successfully sequenced [27]. Variations in genome size among different species may influence the composition and number of MADS-box genes. Unlike *Arabidopsis thaliana*, *Brassica* plants have experienced a complex evolutionary history, including a major genome triplication event approximately 13 to 17 million years ago. This event was instrumental in shaping the unique characteristics that distinguish these species [27]. The genomic data provide a valuable resource for comparative genomic studies. Analyzing MADS-box genes in Chinese cabbage will facilitate research on the molecular mechanisms underlying flowering and further clarify the functions of these genes.

In this study, 102 MADS-box genes were identified and categorized into nine phylogenetic subgroups. Using the MEME [30] tool, ten conserved motifs were identified in Chinese cabbage MADS-box proteins, and four conserved domains were detected, consistent with those in other species. The MADS-box genes were evenly distributed across 10 chromosomes. Numerous collinear blocks were observed between the genomes of Chinese cabbage and *Arabidopsis thaliana*. Interestingly, the total number of MADS-box genes in Chinese cabbage was notably less than three times that of *Arabidopsis thaliana*, indicating gene loss during polyploid speciation.

RNA-seq data from various vernalization stages revealed that many MADS-box genes are regulated by vernalization. Among these, *BrAGL27* was found to be vernalization-responsive, with its expression gradually decreasing as vernalization progressed, suggesting it as a candidate gene for flowering regulation. Further analysis of *BrAGL27* expression through qRT-PCR confirmed a dynamic pattern, with expression initially decreasing and then increasing over the course of vernalization. This indicates that *BrAGL27* plays a role in the vernalization process in Chinese cabbage.

**Figure 8:**
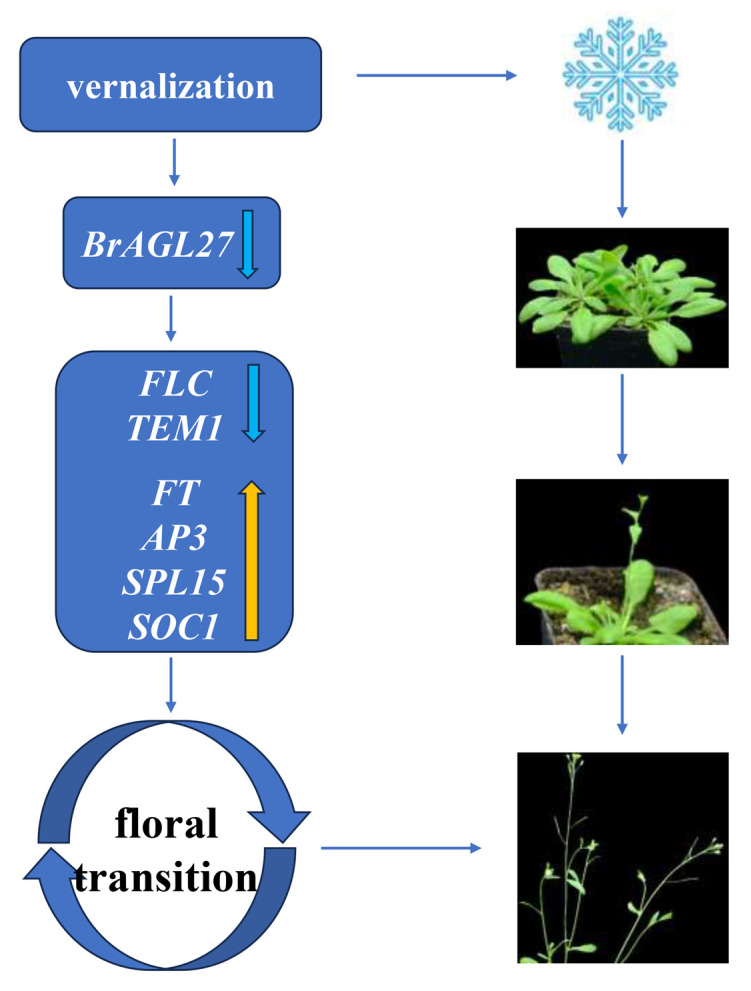
Schematic diagram of the influence of *BrAGL27*’s response to vernalization and its interactions with genes involved in flowering in *Arabidopsis thaliana* plants. A blue arrow indicates a decrease in expression, and a yellow arrow indicates an increase in expression.

To further explore the function of *BrAGL27*, a heterologous overexpression experiment was conducted in *Arabidopsis thaliana*. The overexpression of BrAGL27 in *Arabidopsis thaliana* demonstrated a clear inhibitory effect on flowering. The overexpression lines showed delayed flowering compared to wild-type (WT) plants, with bolting occurring approximately seven days later. Additionally, transgenic plants produced more rosette leaves than WT plants, indicating that *BrAGL27* overexpression effectively suppressed flowering. *AtFLC* and *AtTEM1*, known flowering suppressor genes [29,30], were significantly high-regulated in the overexpression lines compared to WT plants. *FLC* is a key regulator of flowering, serving as the primary suppressor in the vernalization pathways [35]. Both *SOC1* and *SPL15* are involved in promoting flowering and floral meristem determinacy [36,37]. In Pak choi (*Brassica rapa* ssp. *chinensis*), *BcTEM1* promotes flowering [38]. Interestingly, the expression levels of flowering suppressor genes *AtFLC* and *AtTEM1* were significantly high-regulated in the BrAGL27-OE lines compared to WT plants, while the expression levels of the floral genes *AtSPL15, AtSOC1*, *AtFT*, and *AtAP3* were significantly lower in the BrAGL27-overexpressed lines than in the wild type. Therefore, it is highly probable that BrAGL27 inhibits the flowering process of Chinese cabbage by suppressing key floral genes, including *FT*, *SOC1*, *SPL15*, and *AP3*, while simultaneously promoting suppressor genes such as *FLC* and *TEM1*. (Figure 8). All in all, these findings suggest that *BrAGL27* suppresses flowering by influencing the expression of these key regulatory genes.

## 4. Materials and Methods

### 4.1. Plant Materials and Growth Conditions

This study employed the bolting-resistant doubled haploid (DH) line “Ju Hongxin” (JHX) of Chinese cabbage for germination and seedling treatment [39], alongside Colombian wild-type (Col) *Arabidopsis thaliana*. Approximately 400 uniform and healthy JHX seeds were carefully selected, surface-sterilized with water, and placed in Petri dishes lined with two layers of filter paper. The seeds were divided into six groups of 20 seeds each and subjected to the following treatments: non-vernalization and vernalization for 5, 10, 15, 20, and 25 days. Vernalization was conducted at 4 °C, while non-vernalized seeds were maintained at 25 °C. All other culture conditions were kept consistent and optimized to meet experimental requirements.

Non-vernalized seeds were germinated until the seed coat was broken, after which they were transferred to nutrient soil. Plants were cultivated in a controlled greenhouse environment under a 10/14 h light/dark photoperiod with a light intensity of 150 µmol m^−2^ s^−1^. After 30 days, roots, stems, leaves, and shoot apices were collected and immediately frozen in liquid nitrogen for subsequent analysis.

*Arabidopsis thaliana* seeds were sterilized using 75% ethanol, 2% sodium hypochlorite, and sterile water, then plated on Murashige and Skoog (MS) medium. After 10 days of growth, seedlings were transferred to nutrient soil and grown under the same conditions as Chinese cabbage.

### 4.2. Sources of Data and Plant Materials

The complete genome sequence and annotation files for Chinese cabbage were obtained from the Brassicaceae Database (BRAD), specifically using the *Brassica*_Chiifu_V3.0 version [40]. Similarly, the genome sequence and annotation files for *Arabidopsis thaliana* were sourced from the *Arabidopsis thaliana* Information Resource (TAIR). Data on *Arabidopsis thaliana* MADS-box genes were also retrieved from the TAIR database (https://www.arabidopsis.org, accessed on 17 August 2024) [41].

### 4.3. Identification and Analysis of MADS-Box Genes in Chinese Cabbage

The MADS-box protein sequences from *Arabidopsis thaliana* were used as query sequences to identify members of the MADS-box gene family in Chinese cabbage. Sequence alignment was performed using the Basic Local Alignment Search Tool (BLAST) within the TBtools software (v 2.154) package, with an E-value threshold of 1 × 10^−5^ to retrieve candidate Chinese cabbage MADS-box genes. The identified sequences were further validated against the NCBI database using the protein explosion function and screened for conserved domains through the CD Search and Pfam databases. To ensure accuracy, incomplete or redundant sequences were excluded during the analysis. Further, we used the TBtools program to predict the 102 Chinese cabbage MADS-box protein physiochemical characteristics.

### 4.4. Chromosomal Localization and Gene Duplication Analysis of Chinese Cabbage MADS-Box Genes

The amino acid sequences of Chinese cabbage MADS-box genes were mapped onto chromosomes based on genome annotation data, and their chromosomal locations were determined. A visual chart was generated to display the physical positions of these genes. MCscanX [42] was used to identify gene duplication events within the Chinese cabbage MADS-box gene family. Additionally, the Dual Systematic Plot function in the TBtools software (v 2.154) package was employed to visualize collinearity between the genomes of Chinese cabbage and *Arabidopsis thaliana*, highlighting the syntenic relationships of MADS-box genes.

### 4.5. Phylogenetic Analysis, Classification, and Structural and Motif Analysis of MADS-Box Genes

The MEME Suite web server (https://meme-suite.org/meme/, accessed on 23 August 2024) was used to identify motifs within MADS-box genes. The motif distribution was configured to ZOOPS (Zero or One Occurrence Per Sequence), and the number of motifs was set to 10. Statistical analysis of cis-elements was conducted, and TBtools software (v 2.154) was used to visualize the composition of conserved domains, exons, introns, motifs, and promoter regions in MADS-box genes.

Phylogenetic analysis of MADS-box proteins in Chinese cabbage and other species was performed using the neighbor-joining method in the MEGA 11 software package [43] (www.megasoftware.net, accessed on 2 September 2024). Amino acid sequence alignment was carried out using the Maximum Likelihood method with a bootstrap value of 1000 for reliability. All other parameters were set to default. This analysis facilitated the classification of MADS-box genes and provided insights into their structural and functional diversity.

### 4.6. Gene Cloning and Vector Construction

RNA from “JHX” and *Arabidopsis thaliana* were extracted using the RNA simple Total RNA Kit (TransGen Biotech, Beijing, China). With 1 µg RNA as a template, *EasyScript^®^* Reverse Transcriptase was used to complete reverse transcription and obtain the required template cDNA. The obtained cDNA was used as a template for gene cloning, vector construction, and qRT-PCR. The cDNA fragments of these genes were inserted into the pEASY-Blunt vector and the sequences were confirmed by Sanger sequencing. These were then amplified with primers and homologously recombined on digested vectors. All primers in this study used are listed in Supplemental Table S3.

### 4.7. Quantitative Real-Time PCR (qRT-PCR)

Primers for qRT-PCR analysis were designed using Primer Premier 6 and validated through BLAST searches on BRAD (http://www.brassicadb.cn, accessed on 17 August 2024) and TAIR (https://www.arabidopsis.org, accessed on 17 August 2024). The qRT-PCR analyses were conducted on a CFX96 Real-Time System (BIORAD, California, America) under the following conditions: 40 cycles of 95 °C for 10 s, 60 °C for 20 s, and 95 °C for 15 s. Primer specificity was confirmed through melting curve analysis. Relative gene expression levels were calculated using the 2^−ΔCT^ method [44], and one-way ANOVA followed by Dunnett’s multiple comparisons test was performed using GraphPad Prism version 10.0.0 for Windows, GraphPad Software, Boston, Massachusetts USA, www.graphpad.com, accessed on 25 August 2024.

### 4.8. Subcellular Localization Analysis

For the subcellular localization of BrAGL27 proteins, the coding sequence (CDS) of *BrAGL27* (excluding the stop codon) was cloned into the pCAMBIA2300 vector, fusing *BrAGL27* with the green fluorescent protein (GFP) tag. The construct was then transformed into the *Agrobacterium tumefaciens* strain GV3101. *Agrobacterium tumefaciens* cultures containing 35S::*BrAGL27*-GFP and a membrane marker (mCherry protein) were mixed at a 1:1 ratio (800 µL:800 µL) and injected into one-month-old tobacco (*Nicotiana benthamiana*) leaves.

As a control, *Agrobacterium tumefaciens* carrying the pCAMBIA2300 empty vector and the membrane marker (mCherry protein) was similarly injected into one-month-old *Nicotiana benthamiana* (tobacco) leaves at the same ratio. After 48 h of incubation, GFP and mCherry fluorescence in the epidermal cells of tobacco leaves were observed and imaged using a confocal laser scanning microscope (LSM780, Zeiss, Oberkochen, Germany).

### 4.9. Development of Overexpression Lines

The cDNA of *BrAGL27* was cloned into the pCAMBIA1305 vector through homologous recombination. The recombinant plasmid pCAMBIA1305-BrAGL27 was introduced into the *Agrobacterium tumefaciens* strain GV3101 using the freeze–thaw method [45]. Transgenic *Arabidopsis thaliana* plants expressing *BrAGL27* were generated via the Agrobacterium-mediated floral dip method [46].

Seeds from T0 to T2 generations were screened on Murashige and Skoog (MS) medium supplemented with 50 mg·L^−1^ kanamycin. Positive seedlings were further verified by PCR and qRT-PCR, and homozygous lines with single-copy insertions of pCAMBIA1305-BrAGL27 were obtained for downstream analysis.

### 4.10. Statistical Analysis

The data were analyzed for variance, and the means were compared using the student’s *t*-test at a 5% significance level, using GraphPad Prism version 9.5.1. The findings are shown as mean ± standard deviation, with the following significance values: * *p* ≤ 0.05; ** *p* ≤ 0.01; *** *p* ≤ 0.001; **** *p* ≤ 0.0001; and non-significant (ns) (*p* > 0.05).

## 5. Conclusions

In our study, we conducted comprehensive bioinformatics analyses of the 102 genes of the Chinese cabbage MADS-box gene family, including phylogenetic relationships, chromosomal localization, and gene structure. Through real-time PCR quantitative analysis and RNA-seq analysis, it was found that the expression level of *BrAGL27* decreased with the increase in the vernalization time, and its expression levels in the stems, leaves, and shoot apices were relatively high. The subcellular localization of BrAGL27 is on the cell membrane. The heterologous overexpression of *BrAGL27* in *Arabidopsis thaliana* revealed that the expression levels of flowering-promoting genes such as *AtSPL15, AtSOC1*, *AtFT*, and *AtAP3* in the overexpression lines were significantly lower than those in the wild type, while *AtFLC* and *AtTEM1* in the overexpression lines were significantly higher than those in the wild type. These findings provide a detailed understanding of the MADS-box gene family in Chinese cabbage and highlight the role of *BrAGL27* in vernalization responses, offering a foundation for further research into the mechanisms of bolting and flowering.

## Data Availability

Data are contained within the article and Supplementary Materials.

## Supplementary Materials

The following supporting information can be downloaded at: https://www.mdpi.com/article/10.3390/ijms26062635/s1.
