# Supplementary material for: Identification of the MADS-Box Gene Family and the Key Role of BrAGL27 in the Regulation of Flowering in Chinese Cabbage (Brassica rapa L. ssp. pekinensis)
Source: Int J Mol Sci. 2025 Mar 14;26(6):2635. doi: 10.3390/ijms26062635 (PMC11941923; doi:10.3390/ijms26062635)
Supplement: Supplementary file 1 [file ijms-26-02635-s001.zip › ijms-3459786-supplementary.pdf]

Supplemental Table S1. Chinese cabbage *MADS-box* gene family.

Gene ID	Location	CDS (bp)	Protien (aa)
BraA01g001100.3C	514988-517974 (+)	690	229
BraA01g004040.3C	1964213-1966977 (+)	723	240
BraA01g010430.3C	5372075-5372816 (+)	378	125
BraA01g013570.3C	7138703-7141841 (-)	666	221
BraA01g015320.3C	8120215-8126066 (-)	2259	752
BraA01g016140.3C	8578834-8581765 (-)	651	216
BraA01g044460.3C	29320513-29322414 (+)	759	252
BraA02g003340.3C	1616321-1619579 (-)	591	196
BraA02g008770.3C	4130306-4132309 (+)	453	150
BraA02g018970.3C	10736717-10740294 (-)	771	256
BraA02g020340.3C	11755929-11758637 (+)	636	211
BraA02g036180.3C	25159296-25161391 (+)	783	260
BraA02g039100.3C	27229792-27233620 (+)	717	238
BraA02g042570.3C	29545903-29547928 (-)	777	258
BraA02g043090.3C	29859315-29862193 (-)	657	218
BraA02g043490.3C	30117924-30120700 (+)	630	209
BraA02g044910.3C	30859450-30863265 (+)	537	178
BraA02g044920.3C	30871188-30875250 (+)	600	199
BraA02g044940.3C	30880663-30885462 (+)	600	199
BraA03g004170.3C	1785605-1788549 (-)	594	197
BraA03g007090.3C	3038580-3040952 (-)	687	228
BraA03g009520.3C	4080352-4082378 (+)	627	208
BraA03g015270.3C	6812190-6815162 (+)	660	219
BraA03g015280.3C	6822268-6824427 (+)	660	219
BraA03g015950.3C	7336775-7338542 (+)	465	154
BraA03g015960.3C	7341012-7342857 (+)	294	97
BraA03g023780.3C	11414757-11417103 (+)	774	257
BraA03g023790.3C	11418754-11421140 (-)	642	213
BraA03g027090.3C	13417159-13418972 (-)	696	231
BraA03g032820.3C	16290233-16290751 (-)	519	172
BraA03g043550.3C	21987241-21989664 (-)	771	256
BraA03g043880.3C	22193761-22196476 (-)	726	241
BraA03g044420.3C	22454281-22457052 (+)	663	220
BraA03g048590.3C	24677713-24681780 (+)	756	251
BraA03g050620.3C	25991874-25996334 (-)	660	219
BraA03g051930.3C	26833910-26836518 (-)	654	217

BraA03g052540.3C	27170075-27173432 (-)	684	227
BraA03g058700.3C	31089526-31092605 (-)	723	240
BraA04g001360.3C	872711-874717 (+)	693	230
BraA04g002560.3C	1555221-1557026 (-)	525	174
BraA04g003310.3C	2057479-2059000 (-)	846	281
BraA04g005470.3C	3605799-3607200 (+)	417	138
BraA04g016520.3C	12552714-12554295 (+)	528	175
BraA04g016590.3C	12592341-12595391 (+)	687	228
BraA04g018400.3C	13940232-13940774 (+)	543	180
BraA04g024640.3C	17853469-17854017 (+)	549	182
BraA04g031640.3C	21430368-21432782 (-)	642	213
BraA05g003110.3C	1683657-1686843 (-)	735	244
BraA05g005360.3C	2719949-2721810 (+)	729	242
BraA05g005370.3C	2724055-2726497 (-)	642	213
BraA05g011890.3C	6449280-6449657 (-)	279	92
BraA05g015260.3C	8863632-8864009 (-)	279	92
BraA05g041180.3C	27773960-27776046 (-)	753	250
BraA05g042360.3C	28319884-28320465 (+)	582	193
BraA06g004420.3C	2631927-2632682 (+)	756	251
BraA06g014390.3C	7657044-7659110 (+)	1101	366
BraA06g028520.3C	19743867-19744662 (+)	396	131
BraA06g028530.3C	19751255-19752796 (+)	417	138
BraA06g036210.3C	24345939-24349859 (-)	750	249
BraA06g039170.3C	26096299-26098413 (-)	774	257
BraA07g012310.3C	11815444-11817451 (-)	759	252
BraA07g022820.3C	18307531-18309087 (+)	777	258
BraA07g023600.3C	18756186-18759322 (+)	561	186
BraA07g029500.3C	21773637-21775770 (+)	639	212
BraA07g030470.3C	22288958-22294913 (+)	834	277
BraA07g034100.3C	24276920-24279679 (-)	585	194
BraA07g036110.3C	25355464-25356662 (-)	285	94
BraA07g036120.3C	25357993-25358181 (-)	189	62
BraA07g036750.3C	25679383-25680024 (-)	642	213
BraA07g041100.3C	27770070-27773079 (+)	669	222
BraA08g014140.3C	11778582-11779211 (+)	630	209
BraA08g015190.3C	12512687-12516297 (-)	666	221
BraA08g020120.3C	15433931-15436631 (+)	660	219
BraA08g021780.3C	16468817-16469488 (-)	357	118

BraA08g021790.3C	16471867-16472076 (-)	210	69
BraA08g025030.3C	18132265-18134225 (-)	669	222
BraA08g025700.3C	18515348-18519081 (-)	765	254
BraA09g003930.3C	2451243-2454613 (+)	747	248
BraA09g006670.3C	3834707-3835638 (-)	546	181
BraA09g006770.3C	3909911-3913105 (-)	654	217
BraA09g007170.3C	4109283-4114650 (+)	996	331
BraA09g008050.3C	4620452-4621057 (+)	606	201
BraA09g024120.3C	16149584-16151766 (+)	777	258
BraA09g025840.3C	17395418-17396474 (-)	381	126
BraA09g026650.3C	18032034-18036000 (-)	522	173
BraA09g028450.3C	19463804-19466195 (+)	693	230
BraA09g033850.3C	26463142-26464145 (-)	378	125
BraA09g035110.3C	27803042-27803602 (-)	561	186
BraA09g037210.3C	29429665-29431852 (-)	762	253
BraA09g045310.3C	34648176-34650108 (-)	699	232
BraA09g048000.3C	36189376-36190801 (+)	585	194
BraA09g049010.3C	36714272-36717029 (+)	738	245
BraA09g053450.3C	38803942-38804565 (-)	624	207
BraA09g054680.3C	39569039-39574502 (-)	687	228
BraA09g054750.3C	39616299-39619477 (-)	726	241
BraA09g057810.3C	41123801-41124421 (+)	621	206
BraA09g058720.3C	41579239-41579790 (-)	552	183
BraA10g010460.3C	8607666-8610031 (-)	609	202
BraA10g010470.3C	8614030-8618701 (-)	657	218
BraA10g020330.3C	14758676-14761166 (-)	624	207
BraA10g023780.3C	16410444-16412529 (+)	759	252
BraA10g025090.3C	16967309-16968924 (+)	795	264
BraA10g027720.3C	18122666-18127006 (+)	594	197

Supplemental Table S2. Amino acid composition as well as physical and chemical charachteristics of MADS-box protein.

Gene ID	pI	MW/Da	Instability Index	GRAVY	Aliphatic Index
BraA01g001100.3C	8.89	26614.14	42.11	-0.772	86.81
BraA01g004040.3C	7.17	27544.45	42.88	-0.613	91
BraA01g010430.3C	6.29	14354.06	76.07	-0.975	67.92
BraA01g013570.3C	9.19	25099.69	56	-0.762	83.39
BraA01g015320.3C	9.26	84507.4	45.62	-0.282	90.92
BraA01g016140.3C	9.15	25270.35	65.78	-0.561	86.2
BraA01g044460.3C	8.65	28768.69	41.58	-0.706	79.68
BraA02g003340.3C	8.84	21932.23	39.48	-0.469	101.48
BraA02g008770.3C	5.58	17530.22	47.92	-0.68	82.6
BraA02g018970.3C	8.26	30121.21	64.93	-0.909	76.21
BraA02g020340.3C	8.65	23993.91	61.58	-0.365	91.04
BraA02g036180.3C	8.99	29598.77	50.46	-0.577	82.92
BraA02g039100.3C	8.77	26766.72	57.21	-0.635	86.01
BraA02g042570.3C	8.61	30552.62	66.75	-1.023	76.36
BraA02g043090.3C	8.73	25559.57	65.08	-0.665	89.86
BraA02g043490.3C	9.22	24744.53	44.94	-0.761	90
BraA02g044910.3C	8.66	19843.56	55.62	-0.52	93.03
BraA02g044920.3C	8.77	22917.41	55.39	-0.684	84.27
BraA02g044940.3C	9.52	22796.5	52.69	-0.56	89.25
BraA03g004170.3C	9.08	21644.98	40.16	-0.308	108.38
BraA03g007090.3C	9.02	25970.69	42.48	-0.665	82.54
BraA03g009520.3C	8.81	24050.83	42.98	-0.58	88.17
BraA03g015270.3C	9.47	25458.67	53.49	-0.499	93.01
BraA03g015280.3C	9.15	25149.2	46.77	-0.489	86.76
BraA03g015950.3C	10.09	17014.46	49.03	-0.228	94.94
BraA03g015960.3C	5.83	10860.83	14.55	0.187	124.64
BraA03g023780.3C	9.04	29469.61	53.27	-0.547	78.87
BraA03g023790.3C	9.18	24349.8	49.22	-0.794	72.77
BraA03g027090.3C	9.51	26378.94	44.24	-0.674	83.68
BraA03g032820.3C	4.86	19459.73	51.28	-0.896	60.64
BraA03g043550.3C	6.23	29998.78	69.9	-1.018	78.44
BraA03g043880.3C	9.37	27438.35	49.89	-0.62	88.63
BraA03g044420.3C	6.48	25325.17	54.14	-0.459	91.27
BraA03g048590.3C	9.59	28773.34	57.19	-0.918	70.36

BraA03g050620.3C	9.48	25066.76	56.71	-0.746	87.26
BraA03g051930.3C	8.78	24673.14	55.36	-0.643	82.67
BraA03g052540.3C	5.86	25889.47	62.05	-0.631	87.18
BraA03g058700.3C	7.17	27506.35	46.82	-0.651	85.29
BraA04g001360.3C	6.16	26207.56	40.77	-0.658	82.17
BraA04g002560.3C	7.71	20035.51	56.28	-0.871	79.54
BraA04g003310.3C	5.59	31521.36	63.69	-0.666	80.5
BraA04g005470.3C	6.36	16353.36	49.35	-1.015	83.41
BraA04g016520.3C	5.09	19897.06	67.8	-1.043	78
BraA04g016590.3C	8.97	26332.07	63.48	-0.734	85.13
BraA04g018400.3C	9.92	20489.95	44.61	-0.476	77.44
BraA04g024640.3C	5.76	20762.55	52.42	-0.98	61.1
BraA04g031640.3C	9.31	24346.86	53.12	-0.823	74.65
BraA05g003110.3C	9.12	28005.55	59.86	-0.868	80.7
BraA05g005360.3C	9.27	27600.72	38.82	-0.503	77.31
BraA05g005370.3C	9.16	24399.92	59.72	-0.814	76.01
BraA05g011890.3C	10.01	10543.35	31.34	0.063	98.26
BraA05g015260.3C	9.56	10616.35	35.59	0.037	99.35
BraA05g041180.3C	8.44	28569.56	32.03	-0.676	81.48
BraA05g042360.3C	9.2	22172.52	52.79	-0.679	70.73
BraA06g004420.3C	7.04	28795.3	47.01	-0.697	73.75
BraA06g014390.3C	5.68	41902.37	54.01	-0.649	71.69
BraA06g028520.3C	5.71	14882.13	44.29	-0.262	107.1
BraA06g028530.3C	5.35	15981.4	30.72	-0.53	100.36
BraA06g036210.3C	9.33	28136.43	52.15	-0.625	87.27
BraA06g039170.3C	8.74	29422.31	55.4	-0.714	74.4
BraA07g012310.3C	7.71	29253.21	49.97	-0.757	80.91
BraA07g022820.3C	5.95	28867.22	54.06	-0.817	74.46
BraA07g023600.3C	9.18	20980.47	57.21	-0.749	75.97
BraA07g029500.3C	6.85	24002.74	59.45	-0.416	83.25
BraA07g030470.3C	7.71	32414.86	65.12	-0.906	72.89
BraA07g034100.3C	5.58	23097.02	69.54	-1.122	68.92
BraA07g036110.3C	4.63	10830.41	60.86	-0.237	99.68
BraA07g036120.3C	10.11	7043.46	44.55	-0.131	102.1
BraA07g036750.3C	5.48	24105.14	66.68	-0.754	70.52
BraA07g041100.3C	6.85	26363.38	55.38	-0.569	84.82
BraA08g014140.3C	8.71	24352.57	48.51	-0.836	74.11
BraA08g015190.3C	8.86	25170.78	55.15	-0.66	86.02

BraA08g020120.3C	8.97	25303.32	63.03	-0.556	89.91
BraA08g021780.3C	5.56	13787.36	32.48	-1.027	82.63
BraA08g021790.3C	10.01	7849.15	37.93	-0.109	91.88
BraA08g025030.3C	5.58	25580.03	53.06	-0.638	77.3
BraA08g025700.3C	9.11	29878.98	64.13	-0.893	78.35
BraA09g003930.3C	9.4	27970.09	49.48	-0.737	81.37
BraA09g006670.3C	5.55	21677.3	62.86	-1.101	84.53
BraA09g006770.3C	9.66	25004.83	49.46	-0.548	96.59
BraA09g007170.3C	9.9	38441.25	55.97	-0.607	86.31
BraA09g008050.3C	9.08	22550.78	40.91	-0.892	63.13
BraA09g024120.3C	8.91	29560.42	49.82	-0.735	73.72
BraA09g025840.3C	8.32	14475.73	55.35	-0.283	103.65
BraA09g026650.3C	8.92	19781.68	54.61	-0.707	81.16
BraA09g028450.3C	9.36	26269.76	46.89	-0.666	84
BraA09g033850.3C	6.41	14481.6	49.38	-0.385	81.12
BraA09g035110.3C	8.64	20768.57	47.45	-0.629	71.34
BraA09g037210.3C	7.71	29294.18	50.73	-0.786	79.05
BraA09g045310.3C	8.71	27282.85	47.81	-0.854	82.8
BraA09g048000.3C	5.12	21780.06	54.84	-1.011	70.88
BraA09g049010.3C	9.11	27756.3	55.21	-0.783	79.18
BraA09g053450.3C	9.27	23134.65	34.19	-0.423	72.03
BraA09g054680.3C	9.49	26540.23	58.04	-0.786	88.95
BraA09g054750.3C	5.57	27311.68	59.62	-0.722	85.39
BraA09g057810.3C	9.37	23589.21	45.47	-0.693	71.36
BraA09g058720.3C	9.8	20951.14	61.8	-0.607	65.52
BraA10g010460.3C	9.63	23370.19	43.32	-0.662	81.04
BraA10g010470.3C	8.95	25267.27	53.83	-0.524	94.72
BraA10g020330.3C	9.11	23878.73	42.21	-0.623	85.22
BraA10g023780.3C	8.56	28775.66	41.32	-0.748	80.87
BraA10g025090.3C	9.02	30130.05	58.02	-0.753	84.55
BraA10g027720.3C	6.92	22061.56	38.68	-0.188	112.74

Supplemental Table S3. The primers used in this study.

Primer name	Primer sequence (5’-3’)
AGL27-Q1-F	ATGGCCAAGATTCTCAAGCATTACG
AGL27-Q1-R	TTACTTGAGAAGCGGAAGAGTCTCC
1305-*AGL27*-Q1-F	GACGATGACGATAAGGGATCCATGGCCAAGATTCTCAAGCAT
1305-*AGL27*-Q1-R	AGTCCATGGAGATCTAAGCTTTTACTTGAGAAGCGGAAGAGTCTCC
2300-*AGL27*-Q1-F	GGACAGGGTACCCGGGGATCCATGGCCAAGATTCTCAAGCATT
2300-*AGL27*-Q1-R	GGTACTAGTGTCGACTCTAGACTTGAGAAGCGGAAGAGTCTCC
AGL27-QP-241F	TTGGAAGAGCAGTTCAAGA
AGL27-QP-241R	TGAGAAGCGGAAGAGTCT
BrActin-F	GGAGCTGAGAGATTCCGTTG
BrActin-R	GAACCACCACTGAGGACGAT
AtActin-QP-F	TTGACAATTGATGCAAACAATGACG
AtActin-QP-R	CCATTGCTTAATTCCACGGACAAAC
AtFLC-QP-F	GCTACTTGAACTTGTGGATAG
AtFLC-QP-R	TCGGTCTTCTTGGCTCTA
AtTEM1-QP-F	GTCGAGACCGAGTCACGTAAGC
AtTEM1-QP-R	TCACGGCGATGTCGTAAGAAG
AtSOC1-F	ATCGAGTCAGCACCAAACCG
AtSOC1-R	TTCCTATGCCTTCTCCCAAGAG
AtSPL15-F	TCAGCAGCAACTTCAGACACCA
AtSPL15-R	GCTCCGAGATTTGACTCACAGG
AtFT-F	AGTCCTAGCAACCCTCACCTCC
AtFT-R	CCTGCCAAGCTGTCGAAACA
AtAP3-F	ATCAGCCCTAACACCACAACGA
AtAP3-R	CGTCCAAACACTCACCTAGCCT
